# Serum vitamin C levels and risk of osteoporosis: results from a cross-sectional study and Mendelian randomization analysis

**DOI:** 10.1186/s41065-024-00344-w

**Published:** 2024-11-09

**Authors:** Zhiwen Liu, Zijing Peng, Yelin Zhong, Jianjun Wu, Sicheng Xiong, Wei Zhong, Jiehua Luo, Zhihai Zhang, Hongxing Huang

**Affiliations:** 1 Guangzhou University of Chinese Medicine, Guangzhou, China; 2 The Third Affiliated Hospital, Guangzhou University of Chinese Medicine, NO.261 Longxi Road, Liwan District, Guangzhou, 510378 P.R. China

**Keywords:** Serum vitamin C, Osteoporosis, NHANES, Bidirectional mendelian randomization, Causality

## Abstract

**Background:**

The role of vitamin C as an antioxidant in guarding against osteoporosis in adults is still debated. This research employs both a cross-sectional study and a two-sample bidirectional Mendelian randomization (MR) analysis to explore how serum vitamin C levels correlate with the incidence of osteoporosis among adults.

**Methods:**

In this study, we utilized data from the National Health and Nutrition Examination Survey (NHANES) database for the years 2003–2006, and 2017–2018 to conduct both a cross-sectional analysis and MR to investigate the relationship between serum vitamin C levels and the risk of osteoporosis in adults. We adjusted our analyses for essential demographic and lifestyle variables, and applied logistic regression techniques. Genetic determinants of vitamin C levels were analyzed through MR, using methods like inverse-variance weighted (IVW) and MR-Egger to assess causality. Statistical computations were carried out in R, incorporating visual tools such as restricted cubic spline curves (RCS) and forest plots to clarify the dose–response dynamics and variations across different subgroups. This study was approved by the NCHS Ethics Review Board, and informed consent was obtained from all participants.

**Results:**

In our investigation, we analyzed data from 3,940 participants, among whom 291 were diagnosed with osteoporosis. The logistic regression analysis of serum vitamin C quartiles did not indicate a significant trend. The most adjusted model showed a slight, albeit inconsistent, protective effect in the highest quartile (OR = 0.68, 95% CI: 0.47–0.99, *P* = 0.22). Mendelian randomization, employing methods such as IVW, reinforced the absence of a significant causal relationship between serum vitamin C levels and osteoporosis risk (IVW OR = 1.000, 95% CI: 0.999–1.001, *P* = 0.601).Subgroup analyses, visualized through forest plots and restricted cubic spline (RCS) curves, supported the primary findings, showing no significant effects or interactions between vitamin C levels and osteoporosis risk across different demographic and lifestyle subgroups. The RCS analysis particularly highlighted a lack of significant non-linear relationships between serum vitamin C concentration and the odds of osteoporosis (*P* for nonlinear = 0.840).

**Conclusions:**

The cross-sectional study revealed that higher serum vitamin C levels do not consistently correlate with a reduced risk of osteoporosis. Meanwhile, the Mendelian randomization analysis confirmed that there is no genetic evidence to suggest a causal relationship between vitamin C levels and osteoporosis risk. Recent research highlights the polygenic nature of osteoporosis, with genetic predispositions playing a significant role in disease risk. The relationship between serum vitamin C and osteoporosis requires further research. This suggests the need for further investigation into the connection between vitamin C and bone health.

**Supplementary Information:**

The online version contains supplementary material available at 10.1186/s41065-024-00344-w.

## Introduction

Vitamin C is widely heralded for its powerful antioxidant properties, lifes and its crucial role in supporting the immune system, yet its impact on bone health, specifically in the context of osteoporosis, merits closer examination [1]. Known scientifically as ascorbic acid, vitamin C is essential for collagen synthesis, the foundational protein in bones and connective tissues, suggesting its potential influence on maintaining bone density and structural integrity [2]. Vitamin C plays a critical role in collagen synthesis, which is essential for maintaining bone density and overall bone health. Collagen is the primary structural protein in bones, and Vitamin C is a key co-factor in the enzymatic processes that stabilize and cross-link collagen fibers, thereby contributing to bone strength. In addition, Vitamin C acts as a powerful antioxidant, neutralizing free radicals that can damage bone cells and promoting the overall health of bone tissue. These dual functions suggest that Vitamin C could play a protective role against osteoporosis.

Osteoporosis, characterized by weakened bones and an increased risk of fractures, predominantly affects the elderly and poses significant public health challenges [3]. Despite the well-established benefits of calcium and vitamin D in maintaining bone health, the impact of vitamin C on bone density and osteoporosis prevention has been less clear [4]. Research in this area has shown mixed results: some studies indicate that higher dietary intake of vitamin C correlates with better bone mineral density, suggesting a protective effect, while other studies find little to no relationship between vitamin C levels and bone health [5]. These conflicting findings point to the need for more rigorous and comprehensive studies that can offer clearer insights into vitamin C's role in bone metabolism.

To address this gap in knowledge, our research utilizes an extensive dataset from the National Health and Nutrition Examination Survey (NHANES) collected during the periods 2003–2006 and 2017–2018. By employing a sophisticated analytical framework that integrates both cross-sectional and Mendelian randomization techniques, our study aims to dissect the direct observational associations and explore the genetic foundations that may link serum vitamin C levels to the risk of developing osteoporosis. This approach not only provides a more thorough investigation of the observational data but also allows us to examine whether the relationships seen might be driven by genetic predispositions, thereby offering insights into potential causal mechanisms.

Through this dual methodology, we seek to clarify whether the antioxidative and collagen-synthesizing properties of vitamin C translate into a tangible benefit for bone health. If a causal link is established, this could significantly influence public health guidelines and dietary recommendations, potentially leading to the development of preventive strategies that incorporate vitamin C as a key component in combating osteoporosis. This study not only aims to contribute to the scientific understanding of nutrient-bone interactions but also to inform clinical practices and public health strategies aimed at reducing the burden of osteoporosis among aging populations.

## Methods

### Participants in NHANES

**Fig. 1:**
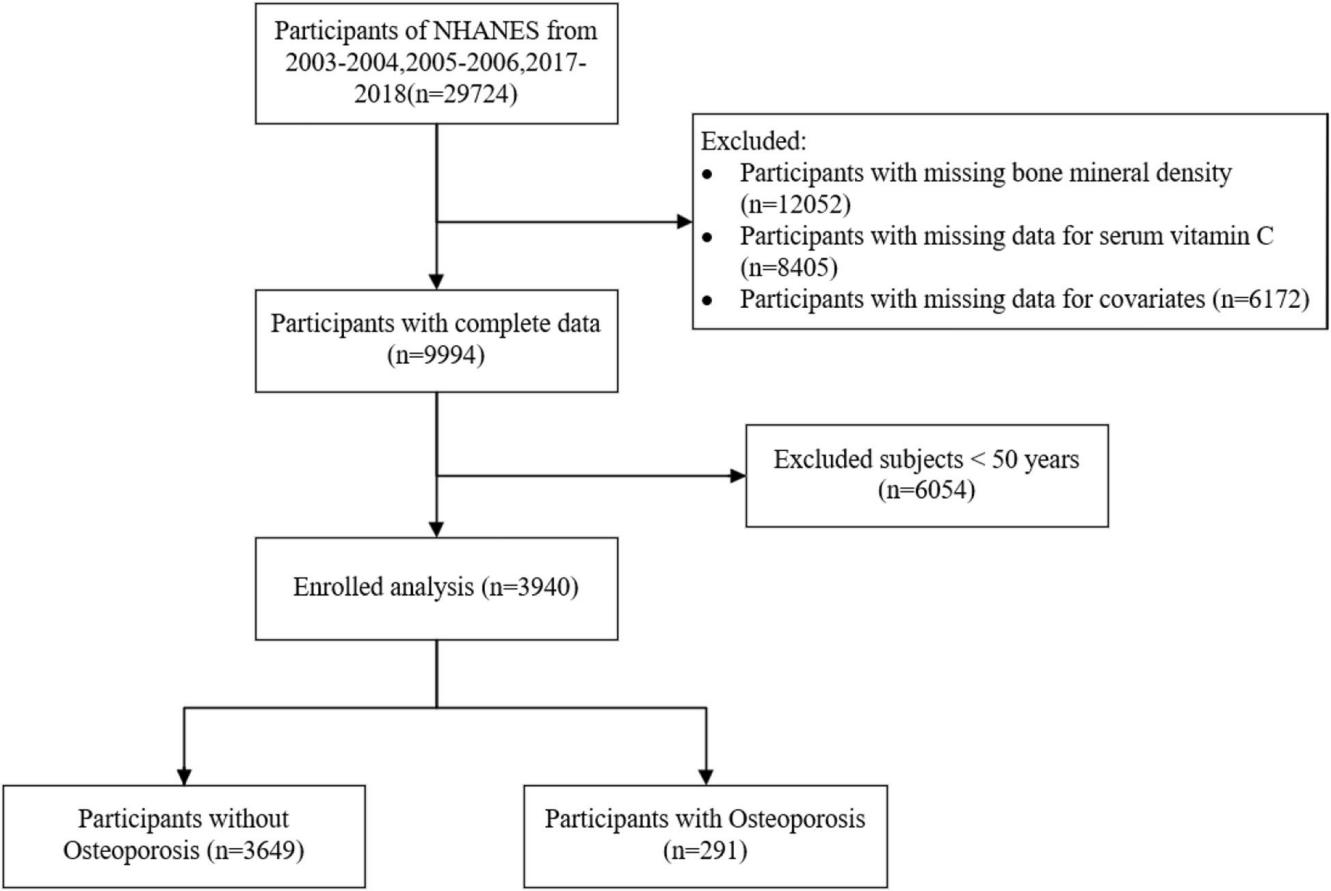
Flowchart of sample selection

In our study leveraging the NHANES data from 2003–2004, 2005–2006, and 2017–2018 (https://www.cdc.gov/nchs/nhanes/index.htm), we initially reviewed 29,724 participants. Through a rigorous exclusion process, we removed individuals lacking critical data: 12,052 for missing bone mineral density, 8,405 for absent serum vitamin C levels, and 6,172 for incomplete covariate information. After refining the dataset to include only those with complete records, 9,994 participants remained. We further narrowed the focus by excluding 6,054 individuals under 50 years old, targeting an older demographic at higher risk for osteoporosis. The final cohort consisted of 3,940 participants, with 291 diagnosed with osteoporosis, providing a robust sample for analyzing the impact of vitamin C on bone health in older adults. Figure 1 illustrates the specific procedure.

### Exposure variables in NHANES

The primary exposure in this study was serum vitamin C levels (µmol/L). To analyze serum specimens, blood samples were meticulously collected, processed, stored, and transported to the Division of Laboratory Sciences at the National Center for Environmental Health in Atlanta, GA, which is part of the Centers for Disease Control and Prevention. The NHANES quality assurance and quality control (QA/QC) procedures adhere to the standards set by the Clinical Laboratory Improvement Act (CLIA). Vitamin C quantification in serum was performed using isocratic ultra-high performance liquid chromatography (UPLC) with electrochemical detection at 450 mV. The range for detection was set at 200 nA. Quantification was achieved by comparing the peak area of vitamin C in the unknown sample to the peak area of a known amount in a calibrator solution. The calibration solution was corrected by comparing the peak area of the internal standard to that of the unknown sample.

## Outcome variable in NHANES

The primary outcome of this study was osteoporosis. Referring to previous studies, dual-energy X-ray (DXA) scans of the spine (L1-L4) or both hip joints were performed to evaluate an individual's BMD (QDR 4500A fan-beam densitometers [Hologic Inc]) [6, 7]. Osteoporosis was defined as a T-score ≤ − 2.5 at either the femoral neck or the lumbar spine. T-scores were calculated as (mean BMD respondent group—mean BMD reference group) / SD reference group. The reference group for calculation of the femoral neck consisted of 20–29 white females from the NHANES III report [6].

## Covariates in NHANES

In our study utilizing NHANES data to assess the effects of serum vitamin C on osteoporosis, we incorporated a variety of covariates to ensure robust and precise analysis [8]. These included demographic details like age, gender, race, and body mass index (BMI); socio-economic indicators such as education, marital status and household income; lifestyle factors like smoking and alcohol use; and medical variables including serum calcium levels, Alanine Aminotransferase (ALT) (U/L), Aspartate Aminotransferase (AST) (U/L), hypertension, and hyperlipidemia. To handle any missing data within these covariates, we applied interpolation techniques, ensuring a complete and comprehensive dataset for our evaluations.

## Sample source for MR analysis

According to previous studies, serum vitamin C’s genome-wide association study (GWAS) information (https://gwas.mrcieu.ac.uk/datasets/ebi-a-GCST90026126/) was obtained from the IEU Open GWAS website (https://gwas.mrcieu.ac.uk/). A total of 291 samples with 6,870,007 singlenucleotide polymorphisms (SNPs) were included in this GWAS data [9]. All sample populations were European, and thus, ethnic variability was excluded. Genetic data about osteoporosis were obtained from the GWAS information (https://gwas.mrcieu.ac.uk/datasets/ebi-a-GCST90038656/) was obtained from the IEU Open GWAS website.This GWAS comprised 484,598 samples in total, with 9,587,836 SNPs [10].

### MR analysis

**Fig. 2:**
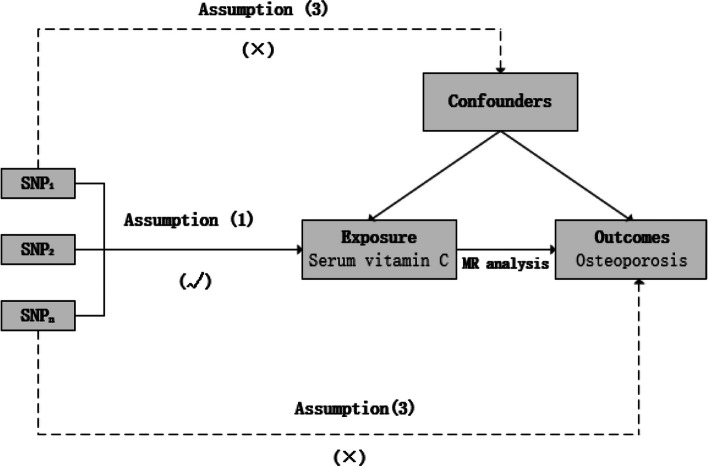
An overview of MR analysis

Figure 2 shows an overview of the present MR study. To obtain valid causal estimates, IVs in the MR model need to satisfy the three assumptions: (1) genetic variants are significantly associated with the serum vitamin C levels; (2) they are not influenced by potential confounders; and (3) they influence osteoporosis only via serum vitamin C levels [7].

We began by identifying genetic variants significantly associated with vitamin C levels, using stringent selection criteria to ensure their suitability as instrumental variables. These criteria included a p-value threshold of less than 5 × 10^–8^, an R^2^ of at least 0.001, and ensuring a genetic distance of less than 10 megabases to minimize linkage disequilibrium [11]. The validity of these instruments was further confirmed through the PhenoScannerV2 tool to exclude any SNPs associated with potential confounders or directly linked to osteoporosis [12]. Details of SNPs for serum vitamin C are shown in Table S1.

We primarily used the Inverse-Variance Weighted (IVW) method for our MR analysis, complemented by additional methods like Weighted median, MR-Egger, Weighted mode, and Simple mode to test the robustness and consistency of our findings [13]. To ensure the reliability of our results, we conducted "Leave-one-out" sensitivity analyses and assessed SNP heterogeneity with the Cochran Q test, considering significant heterogeneity at *P* < 0.05 [14]. We also evaluated horizontal pleiotropy through the MR-Egger intercept, identifying any potential biases at* P* < 0.05.

Furthermore, to bolster our causal inference, we performed a reverse MR analysis using the same stringent methods to explore if osteoporosis might influence serum vitamin C levels [7]. This comprehensive approach ensures that our study's conclusions about the causal relationship between serum vitamin C and osteoporosis are robust, helping to inform potential dietary recommendations and public health strategies targeting osteoporosis prevention.

### Statistical analysis

Quantitative data were analyzed using independent t-tests for normally distributed variables, and Mann–Whitney U tests were utilized for those not meeting normality assumptions, ensuring the appropriate handling of continuous variables. Categorical variables were analyzed using the Chi-square test to assess distribution differences between groups categorized by serum vitamin C levels. Multifactorial logistic regression was utilized in the preliminary analysis to figure out the relationship between exposure factors and outcome indicators. Nonlinear relationships between outcome variables and exposure factors were tested using the Restricted cubic spline (RCS). Subgroup analyses were conducted based on characteristics like age, sex, BMI, smoking, and alcohol consumption. The impact of serum vitamin C on osteoporosis risk across different demographic and lifestyle groups was examined, with odds ratios and 95% confidence intervals calculated for each subgroup. A forest plot was generated to visually summarize the effects of vitamin C across different subgroups. Interaction terms were included in the regression models to test for statistical interactions, ensuring a thorough evaluation of how serum vitamin C's effects might vary across subgroups. For the Mendelian randomization (MR) analysis, we used the TwoSampleMR package in R (version 4.3.2), which enabled us to use genetic instruments for serum vitamin C levels to assess the causal relationship with osteoporosis risk.

All data analyses were performed using the R.4.3.2 procedure (http://www.R-project.org). The available data were used to determine the appropriate sample sizes; ex-ante calculations of sample sizes were not carried out.

## Results

### Baseline characteristics of NHANES participants

As in Table 1, in our study of baseline characteristics using NHANES data, we observed key differences between participants diagnosed with osteoporosis (*n* = 291) and those without the condition (*n* = 3649). Notably, the osteoporosis group was older, with an average age of 66.51 years compared to 62.69 years in the control group, and predominantly female, representing 77% versus 48.3% in the non-osteoporosis group, with both age and gender showing statistically significant differences (*P* < 0.001).The osteoporosis group showed lower educational attainment, with 47.8% not having completed high school, versus 36.7% in the non-osteoporosis group (*P* < 0.001). Additionally, this group was more likely to live below the poverty level (23.7% vs. 14.7%, *P* < 0.001). In terms of health conditions, the prevalence of hypertension was comparable between the groups (63.9% in the osteoporosis group vs. 66.9% in the non-osteoporosis group,* P* = 0.336), whereas hyperlipidemia was slightly more prevalent in the osteoporosis group (23.0% vs. 18.9%), though this difference did not reach statistical significance (*P* = 0.102).

**Table 1:** Characteristics of participants enrolled in NHANES cross-sectional study

Characteristic	Non-osteoporosis (*n* = 3649)	Osteoporosis (*n* = 291)	*P*-value
Age(years)	62.69 ± 9.86	66.51 ± 10.46	< 0.001
Gender			< 0.001
Male	1888 (51.7)	67 (23.0)	
Female	1761 (48.3)	224 (77.0)	
Race			< 0.001
Hispanic	608 (3649)	79 (27.1)	
Non-Hispanic white	1907 (52.3)	151 (51.9)	
Non-Hispanic black	739 (20.3)	18 (6.19)	
Other	395 (10.8)	43 (14.8)	
Level of education			< 0.001
Under high school	1339 (36.7)	139 (47.8)	
High school or equivalent	919 (25.2)	76 (26.1)	
Above high school	1391 (38.1)	76 (25.1)	
Marital status			0.005
Never married	200 (5.48)	15 (5.15)	
Married or living with partner	2315 (63.4)	159 (54.6)	
Divorced, separated, or widowed	1134 (31.1)	117 (40.2)	
Ratio of family income to poverty level			< 0.001
< 1.0	535 (14.7)	69 (23.7)	
1.0 ~ 2.9	1523 (41.7)	131 (45.0)	
≥ 3.0	1591 (43.6)	91 (31.3)	
BMI(kg/m^2^)			< 0.001
< 25	910 (24.9)	124 (42.6)	
25 ~ 29	1102 (30.2)	92 (31.6)	
≥ 30	1637 (44.9)	75 (25.8)	
ALT (U/L)	25.12 ± 35.64	21.17 ± 9.41	0.059
AST (U/L)	25.73 ± 29.53	24.27 ± 8.29	0.400
Serum calcium	9.50 ± 0.39	9.52 ± 0.41	0.390
Drinking			< 0.001
Yes	2629 (72.0)	159 (54.6)	
No	1020 (28.0)	132 (45.4)	
Smoking			< 0.001
Yes	1928 (52.8)	133 (45.7)	
No	1721 (47.2)	158 (54.3)	
Hypertension			0.336
Yes	2440 (66.9)	186 (63.9)	
No	1209 (33.1)	105 ()36.1	
Hyperlipidemia			0.102
Yes	690 (18.9)	67 (23.0)	
No	2959 (81.1)	224 (77.0)	
serum vitamin C (µmol/L)	55.43 ± 30.58	57.05 ± 30.5	0.384

Mean ± SD for continuous variables: the P value was calculated by the weighted linear regression model. (%) for categorical variables: the *P* value was calculated by the weighted chi-square test

Lastly, the mean serum vitamin C levels were slightly higher in the osteoporosis group at 57.05 ± 30.5 µmol/L, compared to 55.43 ± 30.58 µmol/L in the non-osteoporosis group, but this difference was not statistically significant (*P* = 0.384).

### Association of serum vitamin C and osteoporosis

In our investigation, we examined the potential link between serum vitamin C levels and the risk of osteoporosis through logistic regression analysis, categorizing the population into four quartiles based on their vitamin C levels: Q1 (0.6–34.1), Q2 (34.2–55.8), Q3 (55.9–72.7), and Q4 (72.8–268.0). Three different statistical models were applied to account for a variety of demographic and health-related factors. In Model 1, which adjusted for basic demographics such as age, sex, and race, we found no significant decrease in osteoporosis risk with increasing vitamin C levels, displaying odds ratios of [OR:0.95 (95% CI: 0.67–1.36)] for Q2 and [OR:0.77 (95% CI: 0.54–1.11, 0.54–1.09)] for Q3 and Q4, with a P for trend of 0.08 (Table 2). In Model 2, which also considered education, marital status, and income, the association slightly weakened, with [OR:1.02 (95% CI:0.71–1.45)] for Q2, [OR:0.87 (95% CI:0.60–1.25)] for Q3, and [OR:0.88 (95% CI: 0.61–1.26)] for Q4, accompanied by a *P* for trend of 0.40 (Table 2). Our most thorough model, Model 3, included adjustments for BMI, smoking, alcohol consumption, serum calcium, hyperlipidemia, and hypertension. This model showed a notable protective effect of higher vitamin C intake, particularly in Q4, with [OR:0.68 (95% CI:0.47–0.99)] and a *P* for trend of 0.22 (Table 2).

**Table 2:** Odds Ratios for associations between serum vitamin C and osteoporosis

Model	Quartiles of serum vitamin C				*P* for trend
	Q1	Q2	Q3	Q4	
Model 1	1.00	0.95 (0.67–1.36)	0.77 (0.54–1.11)	0.77 (0.54–1.09)	0.08
Model 2	1.00	1.02 (0.71–1.45)	0.87 (0.60–1.25)	0.88 (0.61–1.26)	0.40
Model 3	1.00	1.04 (0.72–1.49)	0.77 (0.53–1.12)	0.68 (0.47–0.99)	0.22

Model 1 was adjusted for age, sex, race

Model 2 was adjusted for age, sex, race, education level, marital status

Model 3 was adjusted for age, race, sex, education level, marital status, smoking, BMI, drinking, serum calcium, ALT, AST,hyperlipidemia, hypertension

**Fig. 3:**
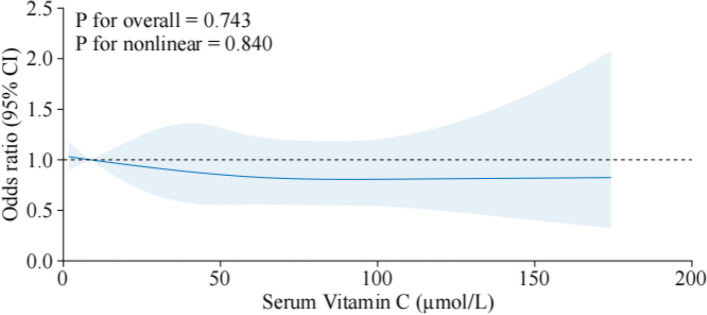
Dose–response relationship between serum vitamin C and osteoporosis. The solid line indicates the estimated risk of osteoporosis, and the dashed line indicates the fitted 95% CI

Our RCS analysis sought to identify potential non-linear dependencies between serum vitamin C levels and the risk of osteoporosis. The outcomes revealed no significant non-linear relationships, with *P*-values for overall effect and non-linearity at 0.743 and 0.840 (Fig. 3), respectively, suggesting a predominantly linear association within the studied range of vitamin C levels.

**Fig. 4:**
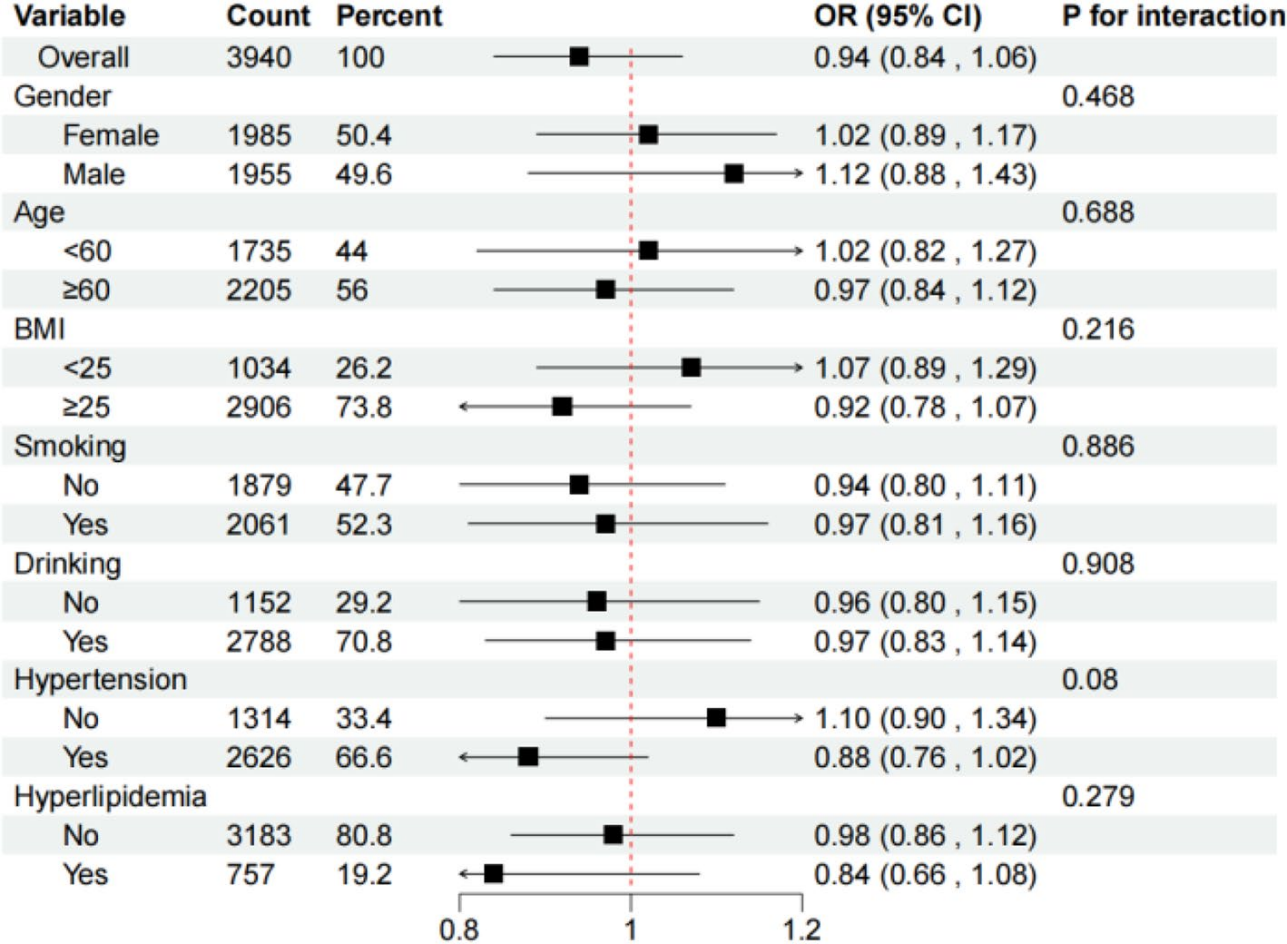
Association between serum vitamin C and osteoporosis in different subgroups. Age, gender, and bmi,smoking, drinking, hypertension, hyperlipidemia were adjusted (the stratified variable was omitted from the model)

Figure 4 shows the subgroup analysis, the forest plot indicated that the protective effect of higher vitamin C levels was more pronounced among non-smokers and those with higher BMI. Specifically, non-smokers with high vitamin C intake showed [OR:0.65 (95% CI:0.42–0.97)], demonstrating a statistically significant reduction in osteoporosis risk, which underscores the interplay between lifestyle factors and nutrient impact.

## The causal relationship between serum vitamin C and osteoporosis

In the previous cross-sectional study, we confrmed a linear negative connection between serum vitamin C and the risk of developing osteoporosis, and to further investigate whether there was a causal relationship, we conducted a bidirectional MR analysis with two samples.

First, we screened 68 SNPs closely related to serum vitamin C (Table S1). Our Mendelian randomization study deployed multiple analytical methods to investigate the potential causal impact of serum vitamin C on osteoporosis risk, and the results uniformly suggest no significant causal association. The Inverse-Variance Weighted (IVW) approach, serving as our primary analysis tool, provided [OR:1.000 (95% CI: 0.999–1.001, *P* = 0.601)] (Table 3 and Fig. S1), indicating that vitamin C levels do not causally affect osteoporosis risk. Further supporting this, the Weighted Median method, known for its robustness even when some genetic instruments might be invalid, also showed a negligible effect with [OR:0.999 (95%CI:0.999–1.001, *P* = 0.874)]. Additional analyses through the Simple Mode and Weighted Mode methods consistently reported ORs around 1.000, with* P*-values of 0.808 and 0.411, respectively, reinforcing the absence of a causal link. The MR Egger method, which adjusts for potential pleiotropic effects of genetic variants, mirrored these findings with [OR:1.000 (95% CI:0.999–1.002, *P* = 0.951)] and an intercept close to zero, further validating the lack of pleiotropic influences. Collectively, these rigorous Mendelian randomization analyses affirm that changes in serum vitamin C levels do not have a causal influence on osteoporosis risk (Table 3).

**Table 3:** Mendelian analysis between vitamin C and osteoporos

Mendelian Method	OR (95%CI)	*P*-value
Inverse-variance weighted	1.000 (0.999,1.001)	0.601
Weighted median	0.999 (0.999,1.001)	0.874
Simple mode	1.000 (0.998,1.001)	0.808
Weighted mode	0.999 (0.998,1.000)	0.411
MR Egger	1.000 (0.999,1.002)	0.951

**Fig. 5:**
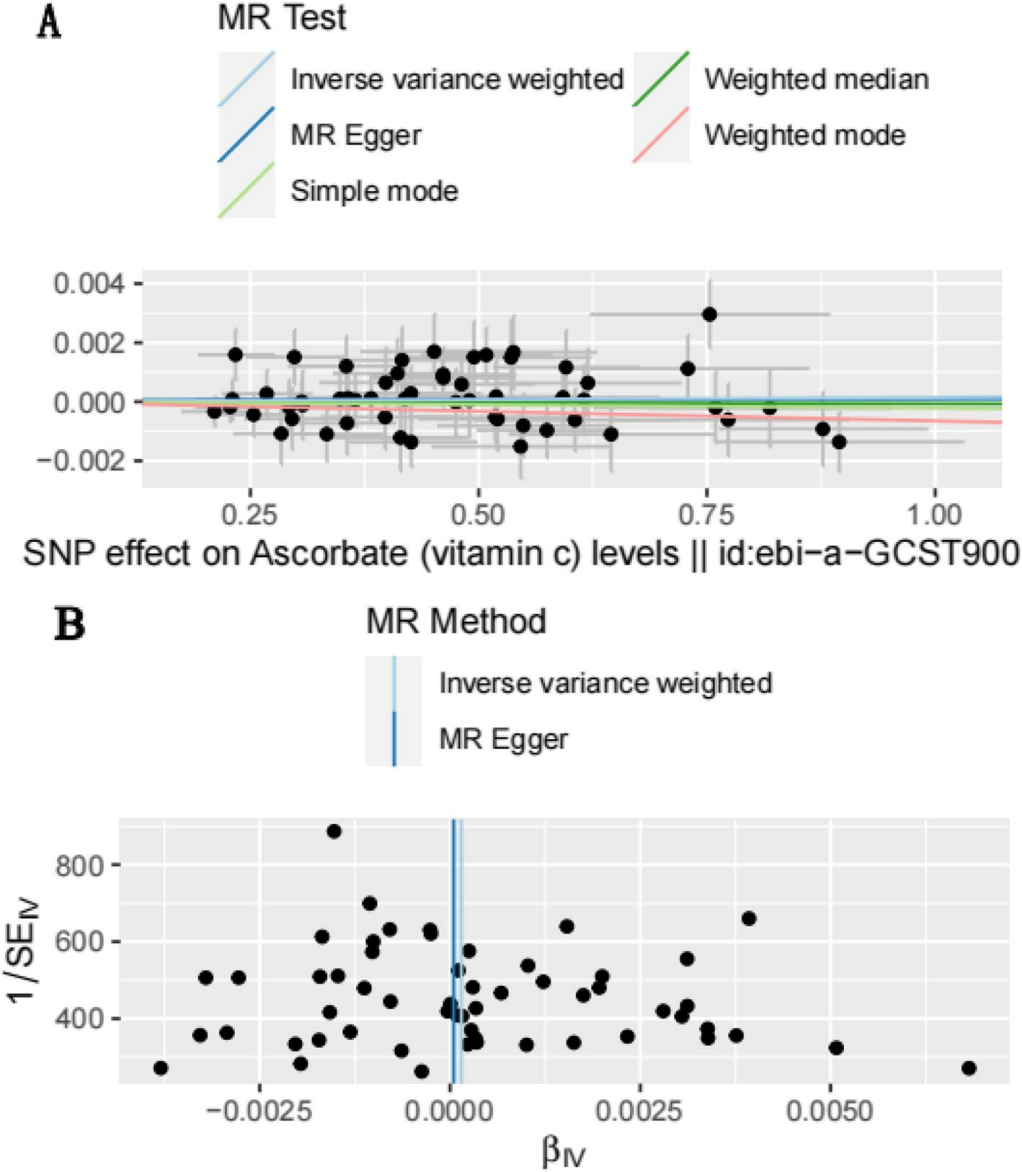
Mendelian randomization analyses the efects of serum vitamin C on osteoporosis. **A** Scatter plot of serum vitamin C on osteoporosis. **B** Funnel plot of serum vitamin C on osteoporosis

Figure 5a presents a scatter plot examining the causal relationship between serum vitamin C levels and osteoporosis risk, evaluated using five different Mendelian randomization (MR) methods. This analysis highlights that the MR-Egger intercept is nearly zero, with the Cochran Q test yielding a value of 46.8 (*P* = 0.745). This suggests a lack of pleiotropic effects and no horizontal heterogeneity across the genetic instruments used in the MR study, pointing to a consistent and reliable assessment.

Additionally, the funnel plot depicted in Fig. 5b shows a symmetrical distribution of the causal effects when individual single nucleotide polymorphisms (SNPs) are utilized as instrumental variables (IVs). This symmetry in the distribution, particularly with 68 SNPs acting as IVs, indicates minimal influence from potential biases, underscoring the robustness and reliability of the findings.

It indicates that the results obtained with 68 SNPs as IVs are less likely to be afected by potential bias, and the results were stable and reliable. Similarly, the outcomes of the “leave-one-out” analysis revealed that after phasing out each SNP, the results of the remaining 67 SNPs analysis were found to be similar to the results of the full inclusion of 68 SNPs analysis, and no SNP loci with strong efects on the results were found in the IVs (Fig. S2). Moreover, a reverse MR analysis explored whether there is an inverse causal relationship between serum vitamin C and osteoporosis risk, as depicted in Fig. S3. This analysis found no evidence of such a relationship, providing additional support for the directionality and integrity of the initial causal findings.

Together, these comprehensive sensitivity analyses affirm the stability and credibility of the results, convincingly demonstrating that variations in serum vitamin C levels do not causally influence osteoporosis risk, based on the genetic data analyzed.

## Discussion

Our comprehensive study combining a cross-sectional analysis and Mendelian randomization (MR) investigates the relationship between serum vitamin C levels and the risk of osteoporosis. This dual approach enabled us to explore not only the direct associations but also the potential genetic underpinnings that could influence these relationships.

The cross-sectional part of our study, leveraging NHANES data, did not find a significant association between serum vitamin C levels and decreased osteoporosis risk across quartiles. Even the most adjusted logistic regression model showed only a slight, inconsistent protective effect in the highest quartile of vitamin C intake (OR = 0.68, 95% CI:0.47–0.99,* P* = 0.22). This finding is in line with research by Kim et al., which reported that while higher dietary intake of vitamin C correlated with better bone health, the effects were not uniformly significant across different populations [15].

These findings align with a systematic review by Malmir et al., which assessed multiple studies and concluded that there is no consistent evidence that higher dietary vitamin C intake is associated with improved bone mineral density or reduced fracture risk across different populations [16]. This suggests that while vitamin C is crucial for collagen synthesis and antioxidant defense, its impact on bone mineral density might be moderated by other nutritional or genetic factor.

Our subgroup analyses, illustrated through forest plots and restricted cubic spline (RCS) curves, highlighted no significant effects or interactions between vitamin C levels and osteoporosis risk across different demographic and lifestyle subgroups, including age, sex, BMI, smoking, and alcohol consumption. This finding is particularly interesting when considering the study by Aghdassi et al., which suggested dietary vitamin C had a more pronounced impact in older adults and smokers due to its role in countering oxidative stress [17]. Krajčovičová-Kudláčková et al. have indicated that higher vitamin C intake is associated with lower levels of oxidative damage in DNA, proteins, and lipids, emphasizing its protective role [18]. Additionally, short-term vitamin C supplementation has been found to significantly reduce lipid peroxidation in smokers, further supporting its antioxidative benefits [19]. Our findings are consistent with Ratajczak’s conclusions on the pleiotropic effects of Vitamin C. Ascorbic acid influences both collagen synthesis and acts as an antioxidant, protecting bone tissue from oxidative damage. Ratajczak also noted that Vitamin C may help prevent osteoporosis, particularly in patients with inflammatory conditions, though the exact mechanisms remain unclear and require further study [20]. The disparity between these findings suggests that other factors, such as overall dietary patterns or concurrent nutrient deficiencies, might play pivotal roles.

The MR component of our study provided more definitive insights, employing multiple methods like IVW, MR-Egger, and Weighted Median. These analyses consistently showed no significant causal relationship between serum vitamin C levels and osteoporosis risk (IVW OR = 1.000, 95% CI:0.999–1.001, *P* = 0.601) (Burgess et al., 2015). This supports findings from a study by Jiaqi Yuan et al., which used similar genetic tools to explore the impact of nutrients on complex diseases, suggesting that the presumed benefits of vitamin C on bone health may not be directly causal [14]. This is consistent with recent genome-wide association studies (GWAS) findings by Semba et al., which suggested that genetic determinants of vitamin C were not directly linked to changes in BMD or the development of osteoporotic fractures [21].

The absence of a significant causal relationship in our study suggests that increasing serum vitamin C alone is unlikely to be an effective strategy for osteoporosis prevention [22]. This aligns with current dietary recommendations by the National Osteoporosis Foundation, which emphasize a balanced intake of multiple nutrients, including calcium and vitamin D, rather than a single-nutrient focus [23].

While our study and other scholarly works like those by Rizzoli et al. and Semba et al. have not established a direct protective role of vitamin C against osteoporosis, they underscore the complexity of nutrient-bone interactions [21, 24]. These findings advocate for a more nuanced understanding that considers genetic factors, dietary patterns, and possibly the synergistic effects of other micronutrients [25].

Calcium Ascorbate is often considered gentler on the gastrointestinal tract and is associated with higher bioavailability due to the slower and sustained release of Vitamin C. However, Ascorbic Acid, which is the more common form, is also effective at increasing serum Vitamin C levels. While there are minor differences in absorption rates, further research is needed to determine the clinical advantages of one form over the other."

The inconsistent findings across studies highlight the potential influence of demographic variables and lifestyle factors, such as age, smoking status, and overall nutritional status, which may modulate the impact of vitamin C on bone health [26]. This complexity is echoed in studies by Aghajanian et al. and Mangano et al., suggesting that vitamin C's effects on bone health could be significant in specific subpopulations, particularly those at greater oxidative stress or with dietary deficiencies [27, 28].

### Strengths and limitations

Our study's insights must be considered in light of several limitations. The cross-sectional design limits the ability to establish causative links, as it captures only a snapshot in time, potentially missing the nuanced long-term impacts of vitamin C on bone health. Furthermore, while the NHANES data set provides a robust sample from the U.S. population, these findings may not extend to global populations with differing dietary patterns and genetic backgrounds [29]. Additionally, serum vitamin C levels, which can fluctuate based on short-term dietary intake, may not accurately reflect long-term nutritional status. Finally, the potential for unmeasured confounders, such as physical activity or sunlight exposure, could skew the relationships observed between vitamin C and bone health outcomes [30]. Addressing these gaps in future research will enhance our ability to craft targeted nutritional strategies for osteoporosis prevention.

To further our understanding of vitamin C's impact on bone health, there is a clear need for longitudinal studies that track changes in bone mineral density over time in response to varied vitamin C intake. Additionally, randomized controlled trials could clarify the potential synergistic effects of vitamin C with essential bone health nutrients like calcium and vitamin D [23]. Exploring genetic and epigenetic influences in greater depth could also shed light on how vitamin C interacts with other metabolic factors at the genetic level, potentially influencing bone health outcomes. Investigating these avenues will provide a more comprehensive understanding of how dietary nutrients, lifestyle choices, and genetic predispositions converge to affect bone health.

## Conclusions

The cross-sectional study revealed that higher serum vitamin C levels do not consistently correlate with a reduced risk of osteoporosis. Meanwhile, the Mendelian randomization analysis confirmed that there is no genetic evidence to suggest a causal relationship between vitamin C levels and osteoporosis risk. Recent research highlights the polygenic nature of osteoporosis, with genetic predispositions playing a significant role in disease risk. This suggests the need for further investigation into the connection between vitamin C and bone health.

## Supplementary Information

Supplementary Material 1.

Supplementary Material 2.

## Data Availability

No datasets were generated or analysed during the current study.

## Abbreviations

NHANES: National Health and Nutrition Examination Survey
MR: Mendelian Randomization
OP: Osteoporosis
SNP: Single Nucleotide Polymorphism
IVW: Inverse-Variance Weighted
IV: Instrumental Variable
RCS: Restricted Cubic Spline
BMI: Body mass indexes
GWAS: Genome-Wide Association Studies
CI: Confidence interval
SD: Standard deviation

## References

[1] Chin KY, Ima-Nirwana S. Vitamin C and Bone Health: Evidence from Cell, Animal and Human Studies. Curr Drug Targets. 2018;19:439–50. 10.2174/1389450116666150907100838. 26343111 10.2174/1389450116666150907100838

[2] Morton DJ, Barrett-Connor EL, Schneider DL. Vitamin C supplement use and bone mineral density in postmenopausal women. J Bone Miner Res. 2001;16:135–40. 10.1359/jbmr.2001.16.1.135. 11149477 10.1359/jbmr.2001.16.1.135

[3] Compston JE, McClung MR, Leslie WD. Osteoporosis Lancet. 2019;393:364–76. 10.1016/s0140-6736(18)32112-3. 30696576 10.1016/S0140-6736(18)32112-3

[4] Sahni S, Hannan MT, Gagnon D, Blumberg J, Cupples LA, Kiel DP, et al. High vitamin C intake is associated with lower 4-year bone loss in elderly men. J Nutr. 2008;138:1931–8. 10.1093/jn/138.10.1931. 18806103 10.1093/jn/138.10.1931 PMC2752366

[5] Rondanelli M, Peroni G, Fossari F, Vecchio V, Faliva MA, Naso M, et al. Evidence of a Positive Link between Consumption and Supplementation of Ascorbic Acid and Bone Mineral Density. Nutrients. 2021;13:1012. 10.3390/nu13031012. 33801019 10.3390/nu13031012 PMC8003869

[6] Hou W, Chen S, Zhu C, Gu Y, Zhu L, Zhou Z. Associations between smoke exposure and osteoporosis or osteopenia in a US NHANES population of elderly individuals. Front Endocrinol (Lausanne). 2023;14:1074574. 10.3389/fendo.2023.1074574. 36817605 10.3389/fendo.2023.1074574 PMC9935577

[7] Zhang K, Shi W, Zhang X, Pang R, Liang X, Xu Q, et al. Causal relationships between COVID-19 and osteoporosis: a two-sample Mendelian randomization study in European population. Front Public Health. 2023;11:1122095. 10.3389/fpubh.2023.1122095. 37293613 10.3389/fpubh.2023.1122095 PMC10244501

[8] Lane JM, Russell L, Khan SN. Osteoporosis. Clin Orthop Relat Res. 2000:139–50.10.1097/00003086-200003000-00016. 10.1097/00003086-200003000-00016 10738423

[9] Panyard DJ, Kim KM, Darst BF, Deming YK, Zhong X, Wu Y, et al. Cerebrospinal fluid metabolomics identifies 19 brain-related phenotype associations. Commun Biol. 2021;4:63. 10.1038/s42003-020-01583-z. 33437055 10.1038/s42003-020-01583-z PMC7803963

[10] Dönertaş HM, Fabian DK, Valenzuela MF, Partridge L, Thornton JM. Common genetic associations between age-related diseases. Nat Aging. 2021;1:400–12. 10.1038/s43587-021-00051-5. 33959723 10.1038/s43587-021-00051-5 PMC7610725

[11] Chen H, Du Z, Zhang Y, Li M, Gao R, Qin L, et al. The Association Between Vitamin C and Cancer: A Two-Sample Mendelian Randomization Study. Front Genet. 2022;13: 868408. 10.3389/fgene.2022.868408. 35601498 10.3389/fgene.2022.868408 PMC9117647

[12] Wang C, Zhang, X. & Qiu, B. Genetically predicted circulating serum homocysteine levels on osteoporosis: a two-sample mendelian randomization study. Sci Rep. 2023;13.10.1038/s41598-023-35472-2. 10.1038/s41598-023-35472-2 PMC10239750 37271768

[13] Liu M, Park S. A Causal Relationship between Vitamin C Intake with Hyperglycemia and Metabolic Syndrome Risk: A Two-Sample Mendelian Randomization Study. Antioxidants (Basel). 2022;11.10.3390/antiox11050857. 10.3390/antiox11050857 PMC9137888 35624721

[14] Yuan J, Peng L, Luan F, Li J, Zhang J, Jiang W, et al. Causal effects of genetically predicted cystatin C on osteoporosis: a two-sample Mendelian randomization study. Front Genet. 2022;13:849206. 10.3389/fgene.2022.849206. 35646051 10.3389/fgene.2022.849206 PMC9136661

[15] Kim J, Yu A, Choi BY, Nam JH, Kim MK, Oh DH, et al. Dietary Patterns Derived by Cluster Analysis are Associated with Cognitive Function among Korean Older Adults. Nutrients. 2015;7:4154–69. 10.3390/nu7064154. 26035243 10.3390/nu7064154 PMC4488778

[16] Malmir H, Shab-Bidar S, Djafarian K. Vitamin C intake in relation to bone mineral density and risk of hip fracture and osteoporosis: a systematic review and meta-analysis of observational studies. Br J Nutr. 2018;119:847–58. 10.1017/s0007114518000430. 29644950 10.1017/S0007114518000430

[17] Aghdassi E, Royall D, Allard JP. Oxidative stress in smokers supplemented with vitamin C. Int J Vitam Nutr Res. 1999;69:45–51. 10.1024/0300-9831.69.1.45. 10052021 10.1024/0300-9831.69.1.45

[18] Krajcovicová-Kudlácková M, Dusinská M, Valachovicová M, Blazícek P, Pauková V. Products of DNA, protein and lipid oxidative damage in relation to vitamin C plasma concentration. Physiol Res. 2006;55:227–31.10.33549/physiolres.930761. 10.33549/physiolres.930761 15910173

[19] Al-Senaidy AM. Effects of short-term supplementation with vitamin C on lipid peroxidation in cigarette smokers. Australian journal of basic and applied sciences. 2010;4(3):487–93.

[20] Ratajczak AE, Szymczak-Tomczak A, Skrzypczak-Zielińska M, Rychter AM, Zawada A, Dobrowolska A, et al. Vitamin C Deficiency and the Risk of Osteoporosis in Patients with an Inflammatory Bowel Disease. Nutrients. 2020;12.10.3390/nu12082263. 10.3390/nu12082263 PMC7468713 32751086

[21] Semba RD, Ferrucci L, Bartali B, Urpí-Sarda M, Zamora-Ros R, Sun K, et al. Resveratrol levels and all-cause mortality in older community-dwelling adults. JAMA Intern Med. 2014;174:1077–84. 10.1001/jamainternmed.2014.1582. 24819981 10.1001/jamainternmed.2014.1582 PMC4346286

[22] Brzezińska O, Łukasik Z, Makowska J, Walczak K. Role of Vitamin C in Osteoporosis Development and Treatment-A Literature Review. Nutrients. 2020;12.10.3390/nu12082394. 10.3390/nu12082394 PMC7469000 32785080

[23] Holick MF. Optimal vitamin D status for the prevention and treatment of osteoporosis. Drugs Aging. 2007;24:1017–29. 10.2165/00002512-200724120-00005. 18020534 10.2165/00002512-200724120-00005

[24] Rizzoli R, Biver E, Brennan-Speranza TC. Nutritional intake and bone health. Lancet Diabetes Endocrinol. 2021;9:606–21. 10.1016/s2213-8587(21)00119-4. 34242583 10.1016/S2213-8587(21)00119-4

[25] Sugiura M, Nakamura M, Ogawa K, Ikoma Y, Yano M. High Vitamin C Intake with High Serum β-Cryptoxanthin Associated with Lower Risk for Osteoporosis in Post-Menopausal Japanese Female Subjects: Mikkabi Cohort Study. J Nutr Sci Vitaminol (Tokyo). 2016;62:185–91. 10.3177/jnsv.62.185. 27465725 10.3177/jnsv.62.185

[26] Carr AC, Lykkesfeldt J. Factors Affecting the Vitamin C Dose-Concentration Relationship: Implications for Global Vitamin C Dietary Recommendations. Nutrients. 2023;15.10.3390/nu15071657. 10.3390/nu15071657 PMC10096887 37049497

[27] Aghajanian P, Hall S, Wongworawat MD, Mohan S. The Roles and Mechanisms of Actions of Vitamin C in Bone: New Developments. J Bone Miner Res. 2015;30:1945–55. 10.1002/jbmr.2709. 26358868 10.1002/jbmr.2709 PMC4833003

[28] Mangano KM, Noel SE, Dawson-Hughes B, Tucker KL. Sufficient Plasma Vitamin C Is Related to Greater Bone Mineral Density among Postmenopausal Women from the Boston Puerto Rican Health Study. J Nutr. 2021;151:3764–72. 10.1093/jn/nxab291. 34510185 10.1093/jn/nxab291 PMC8643605

[29] Lan KM, Wang LK, Lin YT, Hung KC, Wu LC, Ho CH, et al. Suboptimal Plasma Vitamin C Is Associated with Lower Bone Mineral Density in Young and Early Middle-Aged Men: A Retrospective Cross-Sectional Study. Nutrients. 2022;14.10.3390/nu14173556. 10.3390/nu14173556 PMC9459983 36079812

[30] Puspa Zuleika, Legiran. Cross-Sectional Study as Research Design in Medicine. Archives of The Medicine and Case Reports. 2022;3.10.37275/amcr.v3i2.193.

