# Supplementary material for: Serum vitamin C levels and risk of osteoporosis: results from a cross-sectional study and Mendelian randomization analysis
Source: Hereditas. 2024 Nov 9;161:43. doi: 10.1186/s41065-024-00344-w (PMC11549800; doi:10.1186/s41065-024-00344-w)
Supplement: Supplementary file 1 — Supplementary Material 1. [file 41065_2024_344_MOESM1_ESM.docx]

**Supplementary Information**

| 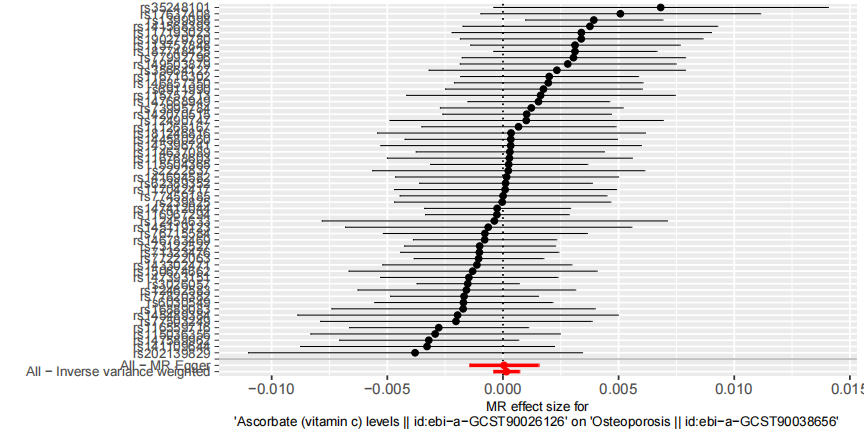 |
| --- |
| **Fig. S1**  Forest diagram of a causal relationship between Serum vitamin C and osteoporosis based on the IVW method. IVW: Inverse-variance weighting |

| 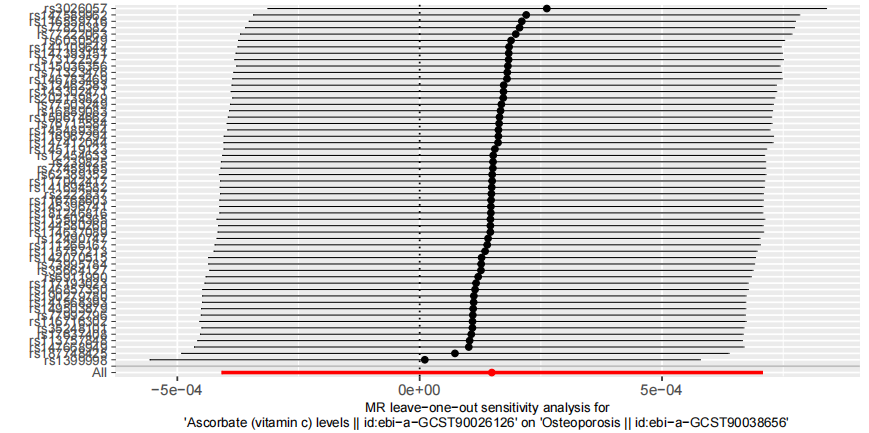 |
| --- |
| **Fig. S2**  [The “leave-one-out” diagram of the causal relationship between Serum vitamin C and osteoporosis](https://static-content.springer.com/esm/art:10.1007/s00223-023-01166-0/MediaObjects/223_2023_1166_MOESM2_ESM.png) |

| 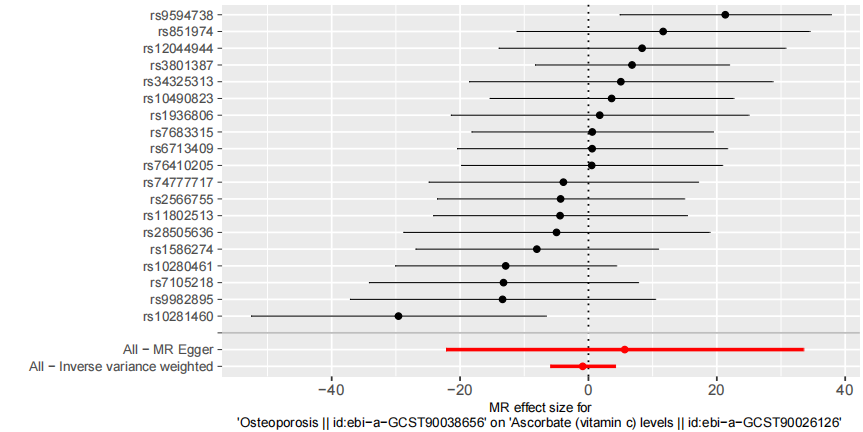 |
| --- |
| **Fig. S3**  Forest diagram of reverse MR analyse between Serum vitamin C and osteoporosis based on IVW method |
